# Changing Sources of Stigma against Patients with HIV/AIDS in the Rapid Expansion of Antiretroviral Treatment Services in Vietnam

**DOI:** 10.1155/2019/4208638

**Published:** 2019-01-21

**Authors:** Bach Xuan Tran, Phung Quoc Tat Than, Tung Thanh Tran, Cuong Tat Nguyen, Carl A. Latkin

**Affiliations:** ^1^Institute for Preventive Medicine and Public Health, Hanoi Medical University, Hanoi, Vietnam; ^2^Bloomberg School of Public Health, Johns Hopkins University, Baltimore, MD, USA; ^3^Institute for Global Health Innovations, Duy Tan University, Da Nang, Vietnam; ^4^California State University, Northridge, Northridge, CA, USA; ^5^Center of Excellence in Evidence-Based Medicine, Nguyen Tat Thanh University, Ho Chi Minh City, Vietnam

## Abstract

Stigmatization against HIV/AIDS greatly hinders efforts to increase the accessibility and utilization of HIV/AIDS services to meet the 90-90-90 goal. This study assessed the stigmatization and discrimination experienced by people living with HIV (PLWH) across multiple social settings such as family, community, and healthcare facilities in Vietnam. A total of 1,016 patients (63.8% males, mean age = 35.4) participated in a cross-sectional study using a culturally tailored HIV stigma measure in three HIV-epidemic-concentrated cities in Vietnam. Zero-inflated Poisson models were used to examine factors associated with the number of types of stigma that patients experienced. 86.2% PLWH reported experiencing stigma against HIV/AIDS, more frequently from their community (62.8%) and family (30.2%) than from health care facilities (8%). The level of stigma from community reported by PLWH is associated with socioeconomic status (e.g., income, occupation). The poor and middle economic classes and unemployed patients reported more stigmatization and discrimination from the community. Across all settings, PLWH experienced fewer stigmatization over the course of ART indicating the benefits of rapidly expanded ART programs. PLWH reported more stigmatization and discrimination at the provincial level of the health administration. Those with the history of drug injection reported significantly less stigmatization from healthcare setting. More culturally tailored interventions to reduce stigmatization overall to improve the quality of life and health outcomes of PLWH should be warranted to achieve the 90-90-90 goal. Improving HIV-related knowledge of the general population and providing opportunities for PLWH to be reintegrated into should be considered. Using mass media with positive messages and images would also foster positive attitudes towards HIV/AIDS among the population and could potentially change social values. Continuous training of health staffs' attitude could minimize the occurrence of stigmatization and discrimination at healthcare facilities.

## 1. Introduction 

Stigma encompasses any stereotypes, prejudices, and unfair treatments of individuals perceived or associated social status, value, or label [1]. It is not naturally occurring but is rooted deeply in culture and is driven by personal and social values. Stigma is a fundamental determinant of health that directly affects patient quality of life and disease treatment outcomes, especially with HIV/AIDS [2]. It has been found in numerous literature that HIV/AIDS-related stigma is the main obstacle to seeking HIV-related services due to feeling shame and the fear of discrimination. Stigma exists in various forms and at different levels such as family, community, and health care sector [3–5]. People living with HIV (PLWH) usually experience self-blaming, social isolation, physical or verbal abuse, mistreating, and political discrimination. This decreases the likelihood of HIV status disclosure, causes adverse health effects such as depression or anxiety, and impacts treatment adherence [6–8]. Injecting drug users (IDU), female sex worker (FSW), and men who have sex with men (MSM) experience double stigma due to their HIV status and their considerably “illegal and immoral” behaviors. HIV/AIDS-related stigmatization experiences differ across cultures and communities. Thus, it is important to understand stigma within a cultural context.

In Vietnam, HIV epidemic concentrated on IDU (9.53%), FSW (2.39%), and MSM (7.36%) [9]. The Government of Vietnam adopted UNAIDS 90-90-90 target which states that 90% of PLWH will know their HIV status, 90% of people with diagnosed HIV infection will receive ART, and 90% of those received treatment will be virally suppressed by 2020 [9]. To respond to this goal, the Vietnamese government has established 401 ART clinics for 124,000 patients by 2018. With the rapid scale-up of ART services, it promises substantial benefits to HIV/AIDS patients in Vietnam. However, HIV/AIDS-related stigma is widespread and remains to be the greatest challenge for LHW to disclose HIV status and seek ART services, which in turn challenges the 90-90-90 goal.

HIV/AIDS-related stigma in Vietnam is perpetuated by the collectivism culture and conservative social values associated with PLWH [10–13]. In the Vietnamese society, people live together as a community and believe in the majority's opinion despite true or false information. From previous national campaign, the negative images of illicit drug users, sex workers, and PLWH had embedded into people's mind as “social evils” [5, 14, 15]. When the majority of people, even health workers, have the prejudice against “social evils” and the fear of infection, PLWH is forced to hide their status and feel ashamed. Their family would receive backlash for having HIV-infected family members. Under social pressure and the fear of infection, HIV-infected individuals are abandoned and expelled from the home [16–20]. Thus, PLWH in Vietnam is afraid of disclosing their HIV status or associating with any HIV/AIDS-related matters due to society's discrimination and stigmatization. As a result, PLWH hardly seeks prevention, treatment, and care services, which make 90-90-90 goal more difficult to achieve.

There have been several studies examining stigma against PLWH in Vietnam in different settings and target groups, including community and family [4], health facility [12], or other social groups [4, 20–22]. However, these studies did not examine the different levels within the health service delivery system. For measurements of stigma, there have been many instruments developed for measuring HIV-related stigma, for example, the PLWH Stigma Index which has been used in many countries, including Vietnam [23, 24]. However, the HIV Stigma Index has a wide breadth of measurement with a long list of items that required intensive resources and patients' collaboration for data collection. In addition, the HIV Stigma Index asked about patient's perception and experience in the past 12 months that might not be effective for use in clinics to monitor patient's well-being over the course of ART. Third, the structure of the HIV Stigma Index was more patient-focused rather than indicating the levels or places in which the stigma exists to facilitate specific interventions to be implemented. Since Vietnam has limited resources, it is important to identify the greatest sources of stigma for targeting with stigma reduction programs; a program may not be been needed in all sectors.

There have been several conceptual frameworks for understanding stigma at multiple levels, including the sociocognitive models at the micro/individual level to the structural models at the macrolevel [25]. In the design of this study, we referred to the theory of structuration by Giddens [26] in which time and space are critical in understanding the agents and structures given the fact that agents' actions will vary in accordance with their contexts [25]. Since stigma against HIV has significantly changed over the past decades as a result of synergic efforts in expanding the coverage of effective interventions, we were interested in identifying the types of stigma experienced by PLWH at different social levels and settings. Therefore, this study assessed stigmatization experienced by PLWH across multiple social domains such as family, community, and levels of healthcare provision. We anticipated that the findings might provide information to assist the Government of Vietnam on how to develop more effective policies, and strengthen efforts to reduce stigma, improve quality of life and health outcomes of PLWH as well as achieve the 90-90-90 goal.

## 2. Materials and Methods

### 2.1. Ethics Statement

The study was approved by the Authority of HIV/AIDS Control. Before enrolling in the study, participants were informed about the objectives, anonymity, and confidentiality of this research. Researchers obtained written consent forms from all participants involved in the study. The confidentiality of the subject information was maintained at all time during the study. Dataset and questionnaire were securely stored.

### 2.2. Settings

In 2012, we conducted the HIV Services Users Survey, which was a cross-sectional study in three cities which have highest HIV prevalence in Vietnam, including Ha Noi, Hai Phong, and Ho Chi Minh City, with the population of 6 million, 1.8 million, and 8 million people, respectively. Three cities were chosen to represent the different geographical areas where has remained the highest HIV prevalence. Ha Noi is the capital city where approximately 18,000 PLWH reside. Hai Phong is a port city in northern Vietnam which has 6,930 PLWH. Ho Chi Minh City is the largest southern metropolitan city with the largest PLWH population, approximately 46,507 people [27]. This survey explored multiple dimensions of the access, utilization, and outcomes of HIV services from the perspective of the patient. Using the same dataset, we have previously published on patient's satisfaction, quality of life, and health care costs [2, 28, 29].

### 2.3. Study Design

ART patients were conveniently selected and invited for an interview when they visited the clinic. A total of 1,016 patients were interviewed; 201 patients (20%) from central level, National Hospital for Tropical Diseases; 406 patients (40%) from provincial level, Dong Da, Viet Tiep, and Ho Chi Minh City Tropical Diseases Hospital; and 409 patients (40%) from district level, Tu Liem, Le Chan, and Binh Tan Health Center.

### 2.4. Study Instrument and Data Collection

Data were collected by researchers and students at the Hanoi Medical University. We did not involve any clinic staff to avoid potential social desirability bias. In addition, we invited patients to a private counseling room to ensure that their information was confidential. During the interview, the researchers collected information using a structured questionnaire. It included socioeconomic status (e.g., age, gender, marital status, education level, occupation, and household monthly income) and self-reported clinical status (e.g., HIV/AIDS stages, Asymptomatic/Symptomatic/AIDS/Unknown, CD4 cell count, history of drug use, and duration of ART). Household monthly income was stratified into five quintiles for analysis purpose.

### 2.5. Measuring Stigma and Discrimination by PLWH

In this study, we developed a contextualized measure to evaluate the stigma and discrimination towards PLWH. First, we reviewed the scope of stigma and discrimination against HIV/AIDS from previously published studies to construct a list of items for measurement. Second, we conducted four focus group discussions with PLWH, their family members, health care workers, and public health researchers for cultural validation and feasibility. Final, the measure was shortened to 20 items. Patients responded “Yes” or “No” for each type of stigma and discrimination that they experienced.

### 2.6. Statistical Analysis

Stata 14.0 software was used for analyzing data. Sociodemographic and HIV-related characteristics of respondents were described using frequency, mean, and standard deviation (SD). Cronbach's alpha was employed to estimate the internal consistency reliability of the stigmatization measures. Factor analysis was utilized to explore the construct of the scale by determining factors and restructuring the items into appropriate factors to increase the interpretability of the measure. Polychoric correlation matrix was applied because each item included in the factor analysis was binary variables. We extracted the factors using the principal component analysis (PCA) with the eigenvalue of 1.0 as a threshold for flattening out the eigenvalue curve and used Orthogonal Varimax rotation with Kaisers' normalization to reorganize the items. We used a value of 0.4 as a cut-off point for factor loadings. Multiple Zero-inflated Poisson regression models were constructed to examine the correlates of the rate of numbers of types of stigma that patients experienced. Candidate independent variables included socioeconomic, drug injection, and HIV-related characteristics of respondents. Statistical significance was defined when the p-value was less than 0.05.

## 3. Results

A total of 1,016 participants were interviewed (63.8% were male). The mean age was 35.4 (SD=7.0). Majority of participants attained education below high school (54.7%) and lived with a spouse or partner (64%). 52.6% of participants were freelancers, and 20.4% had stable jobs. Half of the participants were symptomatic, and one-third of participants (37.6%) had been diagnosed with AIDS. Majority of participants had low CD4 cells count. Only 13.6% participants had CD4 cell count greater than 500 cells/mL. The majority had received ART (88.8%), and 55.3% of those has been treated for at least two years. Most participants reported that they had never used drugs (53.9%).

Types of a stigma that the participants have ever experienced from community, family, or healthcare system or due to HIV status disclosure are presented in Table 1. A majority of participants (82.3%) had told their family members about their HIV status. One-third of the participants (34.5%) reported losing jobs or profits/income due to their HIV status. 34.4% of the participants experienced feared to get HIV infected from them by others. 14.6% of the participants reported being blamed or criticized because they had HIV. 3.6% of the participants perceived receiving poor health care services. 3.1% of the participants reported having been discriminated against by health workers. A majority of participants reported experiencing at least one type of stigma due to HIV disclosure (86.2%); 62.8% from the community, 30.2% from family, and 8% from health care system.

The factors associated with the number of types of HIV/AIDS-related stigmatization that participants have ever experienced are presented in Table 2. These factors are presented across the four domains of community, family, health care system, and HIV disclosure. We found that the number of types of stigma and discrimination by the community was more frequently experienced by patients who were poorer, employed, initiating ART and who were attending clinics at lower levels within the healthcare system.

HIV-related symptoms were also factors that increased the likelihood of experiencing stigma and discrimination by PLWH in families. Symptomatic patients experienced more stigma from family than asymptomatic patients (Coef.=0.69, CI=0.03; 1.36). Meanwhile, those with the history of drug injection experienced less stigmatization from healthcare workers than nonusers (Coef.=-1.92, CI= -2.97; -0.87). In addition, PLWH receiving ART treatment for longer durations experienced less stigmatization and discrimination across settings. For instance, those taking ART between 4-7 years reported less stigma from healthcare workers than those has not taken ART (Coef.=-0.95; CI=-1.99; 0.10).

## 4. Discussion

Our findings contribute the existing literature on how cultural and social values directly affect HIV/AIDS-related stigma and HIV/AIDS care [3, 4, 25, 26]. Overall, the expansion of ART services and the enrollment of HIV patients have reduced stigmatization and discrimination within the community, family, and healthcare settings. However, the stigmatization from the community reported by PLWH remains high, which were associated with different socioeconomic status, employment, ART treatment stage, HIV-related symptoms, and levels of health administration. Interestingly, we found that HIV patients with the history of drug injection reported significantly less stigmatization in the healthcare setting.

For the past decade, the Government of Vietnam has successfully controlled the HIV epidemic and improved quality of life of HIV patients by providing free treatment services [18–20, 28]. Those who initiate ART have to disclose HIV status and will be more likely to feel discriminated by a community when others see them walking to the clinics [24, 25, 30]. As treatment course progresses, HIV patients often obtain better health and are able to manage their life. As a result, ART does not only increase self-efficacy and self-esteem but also improves health outcomes and reduces stigmatization from the community experienced by PLWH [28, 29, 31]. Similarly, because of a collection of effective policy and technical interventions by the Government of Vietnam, with the expansion of Methadone Maintenance Treatment (MMT) facilities and numbers of trained staffs, drug users with or without HIV are receiving additional services. Specifically, those in the sample of this study received various counseling sessions since HIV testing in addition to peer-education and support. Thus, the stigma against drug users and patients with HIV/AIDS has been significantly decreased. Contrary to existing literature, after adjusting to potential confounders in multiple regression models, we found that HIV patients with the history of drug injection experienced less stigmatization in healthcare settings. In previous research conducted in Canada, nurses who constantly worked with illicit drug users developed more positive attitudes and compassion towards this population. Therefore, the more well-trained and specialized staffs may help to reduce stigmatization and make patients feel welcomed which in turn improving treatment adherence and health outcomes of patients.

Despite existing efforts, stigmatization and discrimination remain higher at lower levels of health administration. Staffs at the central level of health administration who receive training more frequently, may perceive and treat HIV patients better. However, at the provincial and district health centers, the staffs, especially general health workers, have less training resulting in poor HIV knowledge that perpetuates prejudicial attitudes towards PLWH. In addition, medical students who have high knowledge and are trained to treat patients professionally reported some misconception regarding HIV transmission and prevention [32]. Some medical students also showed stigmatizing attitudes towards HIV/AIDS by avoiding HIV cases. HIV patients report experiencing nonverbal or verbal discrimination and unfair treatments [11, 33]. For instance, surgeons have refused operating surgery because of the fear of HIV infection, and staffs used different bedding for HIV patients or burned beddings used by HIV patients [10, 33, 34]. These experiences impact HIV patients' decision in seeking medical care in the future.

At the beginning of HIV epidemic era, there were antiprostitution and anti-illicit drug use campaigns that used negatives images to criminalized prostitution and illicit drug use in order to educate the public and stop the spread of HIV/AIDS [7]. However, the campaigns provided insufficient knowledge to cause bias, negative attitudes and fear of HIV transmission. This tactic has caused communities to label PLWH as “social evils,” criminal, failure and immoral [7, 11, 21]. In the context of Vietnamese culture, people believe the evils deserve consequences for their immoral behaviors. Some people believe that PLWH deserves to suffer in poverty and to have a difficult life.

Our findings indicate a relationship between household economic status and stigmatization, that PLWH who were poorer experienced stigmatization from the community. From previous research, PLWH experienced difficulty in finding jobs and maintain their employment due to their HIV status [11, 21]. PLWH often lose their jobs due to their employers' negative perception about HIV and the fear of being infected through casual contact; those who are self-employed lose customers or business partners [7, 24]. As a result, it is difficult for PLWH to earn money for their living. Moreover, poorer PLWH experienced more stigmatization from the community because they might be causing the financial burden or failing to fulfill the expectation as the bread-maker of the family [11, 21, 23]. Unlike the previous study which found that unemployed HIV-infected individuals reported moderate to severe level of felt-stigma, our result indicates those with stable jobs experienced more stigmatization from the community as these people would have a larger social network and in turn, experience more discrimination from this large network. Regardless of their employment and financial status, PLWH in Vietnam experience multiple stigmatizations from the community due to the perception that they committed a “social evil” and deserve the consequences.

HIV patients also receive more stigma from their family due to HIV-related symptoms. In Vietnamese culture, the family remains the sole means of support for most people. When symptomatic HIV patients disclose HIV status to family members, HIV patients often do not usually receive support, but stigmatization and discrimination [7, 11]. Family members might blame PLWH for contracting the “social evil” disease and having HIV symptoms. Moreover, family members often feel shameful and distressed by the community due to the spreading of rumors and gossips about the PLWH; thus, they keep distant, avoid contact, treat HIV-infected individuals differently or even expel them from the home [11, 24]. Losing family support due to HIV status has negative impacts on treatment adherence and health outcomes [11]. Altogether, stigma creates an adverse effect on HIV patients.

## 5. Implications

Previous authors have analyzed the barriers and facilitators of stigma intervention in various settings [33]. Even though our findings indicate that stigma exists in community and family more often than in health care, it is necessary to have interventions at all levels to eliminate stigma. This principle is also supported by findings from a systematic review by Stangl et al. on interventions to reduce the stigma that highlighted the limitation of current practices which only focused on a single socioecological level and a single domain of stigma [30]. Stigma within the community, family, and health care begin with the negative perception of HIV disease. Therefore, it is necessary to provide correct knowledge and address attitudes towards HIV. Unlike using negative images like the previous propaganda, recommended interventions include public education, posters, and campaigns with the positive message displaying where people gather the most or on multiple media outlets (e.g., TV, radio and social media). Interventions that aim to bring HIV population closer to the community should be considered such as inviting HIV patients to participate in talk shows, sharing stories about stigma to create the emotional connection with the community. One suggestion is to use the messages in the Prevention Access Campaign's called “U=U” (undetectable = untransmittable), which could help to reduce the self-stigma among PLWH and their relatives/partners, as well as increased testing and treatment access [34–36]. Furthermore, instead of excluding PLWH from a community, they need to be reintegrated into the community through opportunities such as jobs or vocational training. It must start with mandating laws to abolish stigmatization and discrimination against PLWH in any forms at the workplace. This does not only protect PLWH from stigmatization but also provides a friendly environment where PLWH can work to secure their income. In addition, by providing vocational training to PLWH, they have the skills to work on their own instead of suffering from unemployment and depending on the financial support of others.

However, providing only economic developmental opportunities is not sufficient; it is essential to provide support and motivation to optimize the outcomes [34–36]. This will not only allow them to receive better treatments and improve health outcomes but also strengthen the bond between individuals and family, friends and society. With the goal of better physical and mental well-being and integrate into the community [37], interventions such as family day could help to bring family and HIV patients together could be implemented; family day is when family members are invited to learn about HIV and inform how well patients are doing in an ART program. This could also involve the family's involvement in the patient's treatment plan to improve adherence.

Last but not least, there is a need for additional policies and intervention to address HIV-related stigma and discrimination at all levels of the health care system. Li et al. report on the effectiveness of using popular opinion leaders in reducing HIV-related stigma and improving HIV testing, treatment, and care in the healthcare setting in China [27]. In Senegal, stigma impact mitigation in combination with increased service linkages has also proved to be effective in delivering services [32]. There is a need for training and workshops for health workers at provincial and district treatment facilities on how to communicate and treat patients fairly to avoid HIV-related stigma and discrimination. This is crucial for HIV-infected individuals to feel welcomed and motivated to come to treatment facilities and receive appropriate health care services.

## 6. Strength and Limitation

This study described in-depth HIV/AIDS stigma across multiple social domains including the community, family, and health care system. The study population was recruited from three epicenters to represent geographic difference and to understand stigma across the nation.

There are limitations to the study that need to be considered when interpreting the results. It was a cross-sectional study and there may have been social desirability bias due to sensitive topics. The perceived fear and discrimination could be exaggerated or underestimated. Moreover, the study only measured HIV patients' perspectives. Future study needs to look at community, family, and healthcare workers' perspectives about HIV and HIV patients, as well as opinions from stakeholders such as HIV-related nongovernment organizations or local authorities. Furthermore, we did not address the issue of homosexuality in this study, which should be warranted in further studies. In addition, despite the acceptable reliability, in order to apply this tool in the common practice, the instrument should be improved to ensure the appropriateness regarding the context, language and logical issues. Lastly, since HIV information is confidential due to Law on HIV, the community-based sampling is not feasible. Therefore, we captured only those accessed health services. Therefore, the convenient sample in this study affects the generalizability. This sample was also limited to patients who discontinue their drug use and hence were provided ART. This group is likely to be less stigmatized than active drug users.

## Figures and Tables

**Table 1:** Factor loading, reliability, and measurement of Stigma.

**Items**	%** Yes**	**Factor Loadings**			
		**Community**	**Family**	**Healthcare**	**HIV disclosure**
Having told family members you have HIV	82.3%				0.46
Losing jobs or profits	34.5%	0.62			
Others feared to get HIV infected from you	34.4%				
Having told friends you have HIV	28.8%				0.81
Others gossiped about your HIV status	28.2%	0.67			
Relatives, friends have grown more distant with you	25.8%	0.58			
Being excluded from community events because of HIV	25.6%	0.61			
Afraid that others disclose your HIV status	15.6%				0.44
Being blamed, criticized because you have HIV	14.6%	0.56			
Devalued, disrespected by others because of HIV	14.3%	0.65			
Having told neighbors, you have HIV	11.0%				0.78
Being abandoned by your spouse	9.2%		0.65		
Hurt, annoyed, or offended by others because you have HIV	8.8%	0.49			
Discriminated by family members	7.9%		0.69		
Dismissed or unable to rent an accommodation because of HIV	6.9%		0.47		
Losing heredity because of HIV	6.9%		0.59		
Health workers feared of getting HIV-infected	5.2%			0.65	
Being threatened	4.5%				
Perceived poorer health service quality	3.6%			0.77	
Discriminated by health workers	3.1%			0.76	
**Number of stigma types reported (mean (SD))**		1.59 (1.80)	0.30 (0.70)	0.12 (0.46)	1.41(0.99)
%** patients reported at least one type of stigma**	93.3%	62.8%	20.2%	8.0%	86.2%
Reliability (Cronbach's alpha)		0.75	0.56	0.66	0.65

**Table 2:** Factors associated with the number of stigma types against HIV/AIDS that patients experienced (zero-).

VARIABLES	**Community**		**Family**		**Healthcare**		**HIV disclosure**	
	Coef.	95%CI	Coef.	95%CI	Coef.	95%CI	Coef.	95%CI
**Income per capita (vs Poorest)**								
Poor	0.28*∗∗*	0.03; 0.54	0.12	-0.66; 0.90	0.73	-0.28; 1.74	-0.02	-0.19; 0.15
Middle	0.20	-0.09; 0.50	-0.04	-0.64; 0.57	-0.25	-1.22; 0.72	-0.06	-0.22; 0.10
Rich	0.13	-0.14; 0.39	0.29	-0.22; 0.80	0.58	-0.48; 1.64	-0.03	-0.19; 0.13
Richest	0.10	-0.19; 0.38	-0.19	-0.75; 0.37	-0.45	-1.52; 0.62	0.03	-0.13; 0.20
**Occupation (vs Unemployed)**								
Free lancer	-0.28*∗∗*	-0.53; -0.04	-0.18	-0.63; 0.28	-0.10	-0.82; 0.62	-0.02	-0.16; 0.13
Stable Jobs	-0.42*∗∗∗*	-0.70; -0.15	-0.58	-1.28; 0.11	0.16	-0.69; 1.02	-0.05	-0.22; 0.12
Other	-0.14	-0.43; 0.15	0.14	-0.64; 0.92	-0.52	-1.46; 0.43	-0.02	-0.20; 0.16
**Gender (Male vs Female)**	-0.04	-0.22; 0.15	-0.13	-0.55; 0.28	-0.08	-0.80; 0.64	-0.07	-0.19; 0.05
**Education (>= High school vs < High school)**	0.06	-0.13; 0.24	-0.02	-0.46; 0.42	-0.30	-0.96; 0.36	0.03	-0.08; 0.14
**Marital status (vs Single)**								
Live with spouse/partner	0.16	-0.11; 0.42	-0.20	-1.03; 0.63	-0.39	-1.11; 0.33	0.01	-0.15; 0.16
Widow/Divorce/Separate	0.16	-0.16; 0.48	0.08	-0.91; 1.06	-0.87*∗*	-1.80; 0.06	0.15	-0.04; 0.33
**Ever inject drug (Yes vs No)**	0.04	-0.14; 0.23	0.28	-1.46; 2.02	-1.92*∗∗∗*	-2.97; -0.87	0.09*∗*	-0.01; 0.20
**HIV stage (vs Asymptomatic)**								
Symptomatic	-0.01	-0.30; 0.28	0.69*∗∗*	0.03; 1.36	-0.14	-1.29; 1.01	0.23*∗∗*	0.05; 0.41
AIDS	-0.15	-0.44; 0.14	0.42	-0.20; 1.03	-0.06	-1.00; 0.88	0.12	-0.06; 0.31
**Duration of ART (vs. Not yet)**								
<=1 yr	0.24*∗*	-0.04; 0.52	-0.06	-0.55; 0.42	-0.12	-1.12; 0.89	0.01	-0.20; 0.21
1; <=2 yr	0.03	-0.26; 0.32	0.15	-0.41; 0.70	-0.40	-1.56; 0.77	0.10	-0.08; 0.29
2; <=4 yr	-0.13	-0.40; 0.14	-0.98*∗∗*	-1.78; -0.17	-0.90	-2.04; 0.24	-0.09	-0.27; 0.10
4; <=7 yr	-0.05	-0.33; 0.24	-0.18	-0.70; 0.33	-0.95*∗*	-1.99; 0.10	-0.04	-0.23; 0.14
**CD4 cell count (<= 200)**								
200< CD4 <= 350	0.12	-0.10; 0.34	0.47	-1.53; 2.48	-0.55	-1.99; 0.89	0.00	-0.12; 0.13
350< CD4 <= 500	-0.04	-0.31; 0.24	0.26	-1.99; 2.51	-1.17*∗*	-2.39; 0.05	-0.07	-0.22; 0.08
> 500	0.27*∗∗*	0.00; 0.55	0.56	-1.62; 2.74	-0.66	-1.59; 0.26	-0.12	-0.29; 0.06
**Level of health administration (vs Central)**								
Provincial	0.39*∗∗∗*	0.13; 0.65	0.53	-0.27; 1.32	0.77	-0.91; 2.45	0.12	-0.03; 0.27
District	0.35*∗∗∗*	0.09; 0.62	0.81*∗∗*	0.08; 1.54	1.15	-0.56; 2.86	0.22*∗∗∗*	0.06; 0.37
**Constant**	0.37	-0.11; 0.85	-1.34	-3.67; 0.98	0.78	-2.17; 3.74	0.05	-0.26; 0.35

Robust ci in parentheses

*∗∗∗* p<0.01, *∗∗* p<0.05, and *∗* p<0.1.

## Data Availability

The data used to support the findings of this study are available from the corresponding author upon request.
